# Global Research Trends of Black Soldier Fly Larvae (*Hermetia illucens*) Meal in Aquaculture From a Scientometric Perspective (2007–2025)

**DOI:** 10.1155/anu/5560332

**Published:** 2026-01-06

**Authors:** Julio Camperio, Carlos H. Carroza-Meza, Jorge Suarez, Daniel Benetti

**Affiliations:** ^1^ Marine Biology and Ecology, University of Miami Rosenstiel School of Marine, Atmospheric, and Earth Science, University of Miami, Miami, Florida, 33136, USA, miami.edu; ^2^ Abess Center for Ecosystem Science and Policy, University of Miami, Coral Gables, Florida, 33146, USA, miami.edu

**Keywords:** aquaculture, bibliometric, black soldier fly larvae, global research, scientometric

## Abstract

Black soldier fly larvae (*Hermetia illucens*) meal (BSFLM) has gained increasing attention over the past two decades as a sustainable and functional ingredient in aquafeeds. This study presents the first scientometric analysis of BSFLM research in aquaculture from 2007 to 2025, using data from Scopus and Web of Science (WOS). Following PRISMA‐guided screening, 355 peer‐reviewed articles were retained and analyzed with the *Bibliometrix* R package. Results indicate a consistent annual growth rate of 11.42% in publications, with Italy, the United States, Norway, and China emerging as key contributors. Research themes have evolved from initial feasibility studies to more recent emphases on health parameters, immunological effects, and gut microbiota modulation. Species such as *Oncorhynchus mykiss*, *Sparus aurata*, *Oreochromis niloticus*, *Salmo salar*, *Dicentrarchus labrax*, and *Litopenaeus vannamei* are frequently studied, reflecting their commercial and academic relevance. However, a pronounced underrepresentation of carps and catfish, despite being the most farmed finfish globally, highlights a persistent misalignment between research priorities and global aquaculture production, likely due to the large variety of regional species being produced and investigated. Further regional disparities exist, with Europe accounting for 50% of the literature but only 3.2% of global output, while Asia accounts for 30% of the literature but 89% of global production output. These findings offer a road map to realign global research priorities with aquaculture production realities.

## 1. Introduction

Over the past two decades, black soldier fly larvae (*Hermetia illucens*) meal (BSFLM) has attracted significant scientific attention as a sustainable protein source for aquaculture [1]. Driven by increasing demand for environmental feed alternatives and the rising costs and ecological impacts associated with fishmeal and soybean meal, BSFLM has emerged as a promising candidate in fish and shrimp diets [2, 3]. Numerous studies have investigated the nutritional value, digestibility, growth performance, and sustainability benefits of incorporating BSFLM into aquafeeds, positioning it as a key innovation in sustainable aquaculture [4–6].

Despite the rapid growth in research activity, the existing literature is fragmented across different species (Atlantic salmon, Nile tilapia, European seabass, whiteleg shrimp, grass carp, and African catfish, among others), experimental designs (as feed additive or full and partial fish meal replacement, different larvae and meal processing methods, varied inclusion levels, different life stages and culture systems such as RAS and flow‐through, and evaluation of different production and health parameters), and regional contexts (Norway, Thailand, Italy, Portugal, China, and Hungary, among others) [6–12]. While individual studies have contributed valuable insights, there has been no systematic mapping of the global research landscape surrounding BSFLM use in aquaculture. Scientometric analyses offer a powerful tool to address this gap by quantitatively examining publication trends, identifying leading countries, institutions, and authors, and highlighting the evolving research priorities within the field.

Scientometric analysis has become an increasingly valuable methodological approach for both academia and industry, offering structured insight into the evolution, productivity, and global dynamics of research fields. As highlighted by Chen and Song [13], systematic scientometric reviews combine bibliometric data with visualization and network analytics to enhance the timeliness, accessibility, and reproducibility of literature synthesis, enabling researchers to map intellectual structures and identify emerging knowledge domains. For academia, such approaches guide research agendas, funding strategies, and collaboration networks by quantifying thematic development and geographic trends [14]. In the aquaculture sector, scientometric analyses have recently been used to assess research productivity and thematic evolution across several disciplines: Raghuvaran et al. [15] mapped global trends on a select novel meal in aquaculture, revealing a rapid growth of publications and highlighting regional and institutional leaders; Aly and Fathi [16] demonstrated the application of scientometrics in biosecurity research, identifying dominant research themes and policy implications for disease prevention; and Meenakshisundaram et al. [17] integrated bibliometric mapping with critical review to analyze mycotoxin‐related research in aquafeed, linking publication patterns with regulatory gaps and regional vulnerabilities. Similarly, Veluman et al. [18] applied scientometric analysis to climate change and fisheries, showing how such tools can trace temporal shifts and emerging environmental priorities. Collectively, these studies underscore the dual importance of scientometric methods by advancing academic understanding of knowledge structures while informing industrial innovation and policy and reveal which technologies, species, or sustainability themes are poised for translation from research to application.

This study aims to provide a scientometric analysis of the global scientific output on BSFLM in aquaculture. By utilizing systematic review methodologies and bibliometric visualization tools, this research seeks to map the intellectual structure, collaboration networks, and thematic developments from 2007 to 2025. Specifically, the objectives of this study are (1) to examine publication trends over time, (2) to identify the most influential countries, institutions, and authors, (3) to visualize international collaboration networks, and (4) to characterize the dominant research topics and their evolution across distinct time periods.

Through this analysis, we aim to offer strategic insights into the development trajectory of BSFLM research, guide future investigations, and support stakeholders, ranging from academics to industry practitioners, in navigating the growing field of insect‐based aquafeeds. This study also aims to highlight underrepresented regions and species, offering recommendations for future research.

## 2. Materials and Methods

The data search was conducted in two of the most relevant databases for scientific papers, collecting data from Scopus and Web of Science (WOS). The search did not specify a time period for analysis, instead covering the entire period available in each database. Queries were performed in both databases using the keywords “*Hermetia illucens*” and “*aquacultur*e.” The searches were conducted on the same day (March 25, 2025) to minimize potential bias from database updates and were performed on article titles, abstracts, and keywords. As a result, a total of 264 articles were retrieved from Scopus and 506 from WOS.

**Figure 1:**
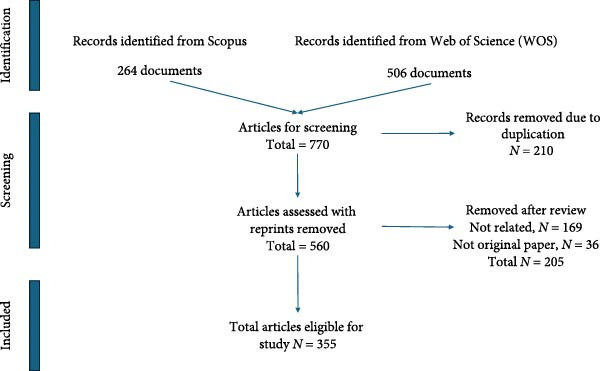
PRISMA systematic scoping review flow process.

Although PRISMA was developed primarily for systematic reviews and meta‐analyses, its principles of transparent reporting are broadly applicable to studies involving structured literature retrieval and selection. Scientometric analyses, such as systematic reviews, require clear documentation of how records are identified, screened, and excluded to ensure replicability and minimize bias. Following the PRISMA 2020 framework, we employed its flow diagram and reporting logic to transparently present the selection process, from initial retrieval in Scopus and WOS to the final inclusion set [19]. This adaptation aligns with the rationale behind extensions such as PRISMA‐ScR for scoping reviews, reinforcing that the framework can be appropriately applied beyond traditional health intervention reviews when the goal is to improve transparency and reproducibility in research synthesis [20]. The PRISMA flow diagram (Figure 1) provides a structured overview of the screening process, detailing the sequential reduction in the number of articles and the specific criteria applied for exclusions.

In the initial screening process, 210 duplicate articles identified across both databases were removed using the function “mergeDbSources” from the package “bibliometrix version 4.3.3” in R. This method has been used to merge and remove duplicate articles from databases such as WOS and Scopus, which resulted in a total of 560 unique articles [21]. This set was then further refined by excluding all documents that did not constitute original, peer‐reviewed research. Specifically, conference papers, literature reviews, and book chapters were excluded, eliminating an additional 36 articles. Subsequently, the titles and abstracts of the remaining articles were reviewed to assess if they are related to the original search criteria, namely, studies focused on *Hermetia illucens* (black soldier fly) within the aquaculture sector. This final screening led to the removal of 169 unrelated articles. As a result, a total of 355 articles were retained for inclusion in the scientometric analysis.

To carry out the scientometric analysis, the R package *Bibliometrix* (4.3.3.) was used to visualize the information and create a Sankey diagram (three‐field plot), which was used to graphically represent the relationship between authors, countries, and sources. At the same time, the package estimated bibliometric indexes such as the annual growth rate (%), average document age, average citations per document, and percentage of international co‐authorship, based on the information provided by the WoS and Scopus databases [22].

The bibliometric analysis included several key indicators to evaluate the evolution, impact, and collaboration patterns of the research field. The annual growth rate (%) measures the yearly increase in the number of published documents, indicating the pace of scientific production over time. The document average age represents the mean number of years since publication of all documents, reflecting whether the literature base is recent or historically established. Similarly, the average citations per document expresses the mean number of citations received by each publication, providing an estimate of the average scholarly influence within the dataset.

Collaboration patterns were assessed through the international co‐authorships (%), which quantifies the proportion of papers co‐authored by researchers from different countries. This indicator highlights the level of global collaboration and the integration of international research networks. Citation performance was further explored using the mean total citations (TCs) per tear (also referred to as TC per Year), which measures the average number of citations a paper receives annually since publication. This normalization allows equitable comparisons between older and newer works, accounting for differences in citation exposure time.

Finally, the normalized TCs (Norm TC) adjusts raw citation counts to account for variations in publication year or disciplinary citation practices, enabling fairer cross‐field and temporal comparisons. Collectively, these metrics provide a comprehensive overview of the productivity, temporal structure, international collaboration, and scientific impact of the research field analyzed through the *bibliometrix* R package [22].

We also used the R platform to analyze the number of species mentioned in the title of each article. We extracted the scientific names of each species using the “stringr” library and wrote a function that captured each word in italics in the title. We additionally reviewed the titles of the articles not identified in the previous step, searching for the scientific and common names of aquaculture species. The lists of species identified by the R script and manual review were then analyzed together.

To carry out the trend analysis of the detected literature, we used “keywords plus” instead of author keywords. Keywords Plus, generated by an automatic computer algorithm, are words or phrases that frequently appear in the titles of an article’s references but not necessarily in the article’s own title or as author keywords [23, 24]. Keywords Plus terms emphasize research methods and techniques more strongly than author keywords [24]. In our analysis, 92.13% of the articles contained Keywords Plus, which provided better quality for the analysis compared to Author Keywords, present in 90.17% of the articles.

## 3. Results

Following the PRISMA analysis, a total of 355 articles were identified spanning the period from 2007 to 2025. These articles, summarized in Table 1, involve contributions from 1847 unique authors. The number of publications has grown at an average annual rate of 11.42%, highlighting a steady and sustained increase in research activity overtime.

**Table 1:** Main information extracted.

Description	Results
Main information about data	
Timespan	2007–2025
Sources (journals, books, etc.)	89
Documents	355
Annual growth rate (%)	11.42
Document average age	3.57
Average citations per doc	33.84
Document contents	
Keywords plus (ID)	1013
Author’s keywords (DE)	911
Authors	
Authors	1847
Authors of single‐authored docs	2
Authors collaboration	
Single‐authored docs	2
Co‐authors per doc	7.77
International co‐authorships (%)	32.68

*Note:* Values calculated used R package Bibliometrix version 4.3.3.

**Figure 2:**
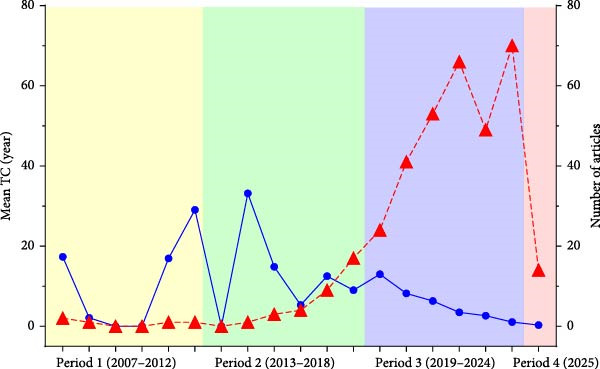
Temporal evolution of scientific publications and mean total citations (TC) per year. The dashed red line indicates the number of published articles; the solid blue line indicates the mean total citations per year.

Figure 2 illustrates the annual progression of both the number of published articles (dashed red line, right *y*‐axis) and the mean TCs per year (solid blue line, left *y*‐axis) from 2007 to 2025. To contextualize the development of the field, the timeline has been divided into four research periods, represented by shaded background colors: Period 1 (2007–2012, yellow), Period 2 (2013–2018, green), Period 3 (2019–2024, blue), and Period 4 (2025, pink).

During Period 1, research activity was minimal, with few publications but relatively high mean citations. Period 2 shows a modest increase in article output and continued variability in citation impact, suggesting growing but still sporadic attention to the topic. A sharp increase in article production is observed in Period 3, particularly between 2019 and 2024, indicating a rapid expansion in scientific interest and output in the application of black soldier fly in aquaculture. This period also shows a general decline in mean TC per year, possibly reflecting a citation lag for newer publications or a diversification of research topics. Period 4, representing only the year 2025, shows a decrease in both article count and mean citations, which is expected due to its recency and limited time for citation accrual.

**Figure 3:**
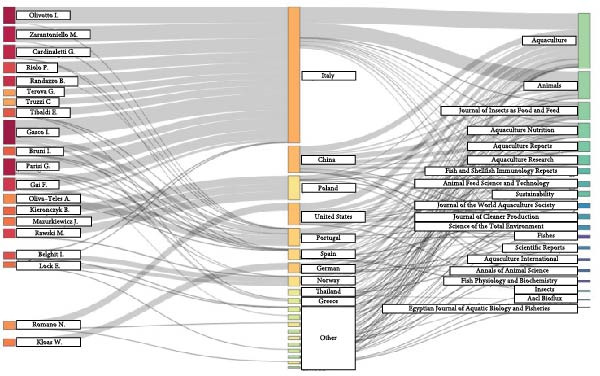
Sankey diagram (three‐field plot) containing the top 20 authors (left field), corresponding authors’ country (middle field), and source (right field).

The Sankey diagram (Figure 3) presents authors on the left nodes, respective country affiliation on the central node, and the scientific journals where their research outputs have been published on the right node. The width of the connecting flows reflects the frequency or strength of the association, providing a visual representation of publication trends, collaborative intensity, and national research output within the field. Notably, a significant proportion of the researchers are affiliated with institutions in Italy, indicating this country’s prominent role in the research domain.

The diagram further reveals the diversity of publication venues, with journals such as *Aquaculture*, *Animals*, and the *Journal of Insects as Food and Feed* receiving substantial contributions. This suggests a multidisciplinary approach encompassing aquaculture nutrition, alternative feed sources, and animal science. The presence of authors from countries including Poland, Portugal, the United States, and China, alongside a variety of international journals, underscores the global nature of the research.

**Figure 4:**
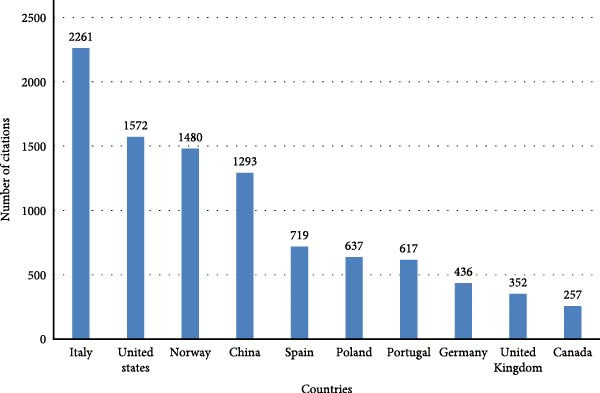
Number of citations received by the top 10 countries.

Figure 4 presents the total number of citations received by the top 10 contributing countries in the field. The bibliometrix R package estimates citations for countries based on the reprint address defined for each publication. This information serves as an indicator of research impact and visibility within the scientific community. The data reveal a marked dominance by Italy, which accumulated 2261 citations, significantly surpassing all other countries. The United States and Norway follow with 1572 and 1480 citations, respectively, reflecting their strong presence in the publication landscape and the influence of their research outputs. China also demonstrates a notable contribution with 1293 citations, affirming its growing role in aquaculture and related disciplines. The remaining countries of Spain, Poland, Portugal, Germany, the United Kingdom, and Canada show a more moderate citation count, ranging from 719 to 257, highlighting a secondary tier of international participation and impact.

This citation distribution aligns with the authorship and journal contribution patterns previously illustrated in the Sankey diagram, where Italy emerged as the most prominent contributor both in number of researchers and publication outputs. The high citation counts of countries such as the United States, Norway, and China further underscore their research relevance and the broader dissemination of their findings. The data suggest that while a wide range of countries contribute to the body of literature in aquaculture science, a few nations, particularly Italy, lead in shaping the scientific discourse through both volume and citation influence.

**Figure 5:**
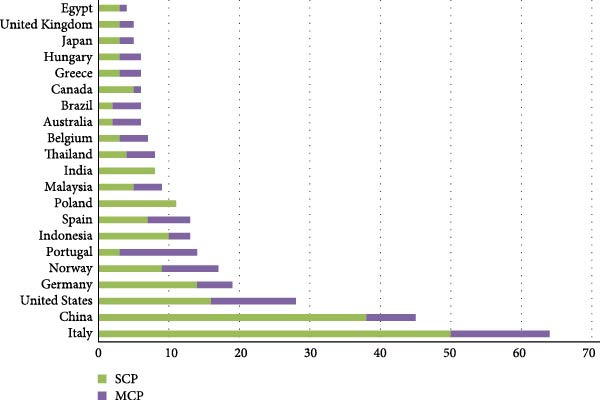
The top 20 nations as presented based on the number of publications. MCP, multiple country publications; SCP, single country publication.

Figure 5 presents a comparative analysis of the top 20 nations based on the number of scientific publications, classified into two categories: single‐country publications (SCP) and multiple‐country publications (MCP). SCP, depicted in green, refers to studies authored by researchers affiliated with institutions from a single nation, while MCP, shown in purple, represents collaborative outputs involving contributors from multiple countries.

Among the countries listed, Italy stands out as the leading contributor in terms of total publications, with a predominant share corresponding to SCP. China follows closely, also exhibiting a high number of SCP, though with a slightly more balanced distribution between SCP and MCP. The United States ranks third overall, characterized by a more pronounced contribution in MCP, indicating a higher degree of international collaboration relative to SCP.

Germany and Norway similarly display substantial contributions, with Germany showing a relatively even distribution between SCP and MCP, and Norway demonstrating a stronger presence in MCP. In contrast, nations such as Poland, Malaysia, and India report a higher proportion of SCP, suggesting a more nationally concentrated research output.

Countries with lower total publication counts, such as Egypt, the United Kingdom, and Japan, are represented with modest values in both SCP and MCP categories. However, even among these nations, the variation in the balance between domestic and collaborative research is evident.

**Figure 6:**
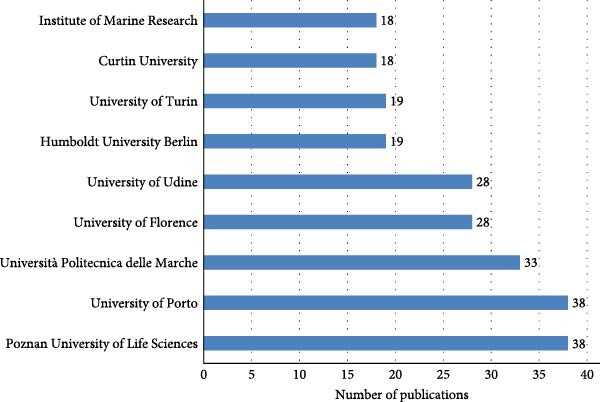
Most relevant affiliation.

Figure 6 displays the 10 most productive institutions in terms of scientific output, as measured by the number of publications. Each bar represents a distinct institution, with the length of the bar corresponding to its total number of publications. The Poznan University of Life Science in Poland and the University of Porto in Portugal lead the ranking with the highest publication counts, both exceeding 35 articles. These are followed closely by the Marche Polytechnic University and the University of Florence, each with more than 25 publications, indicating a strong research presence in these institutions.

**Figure 7:**
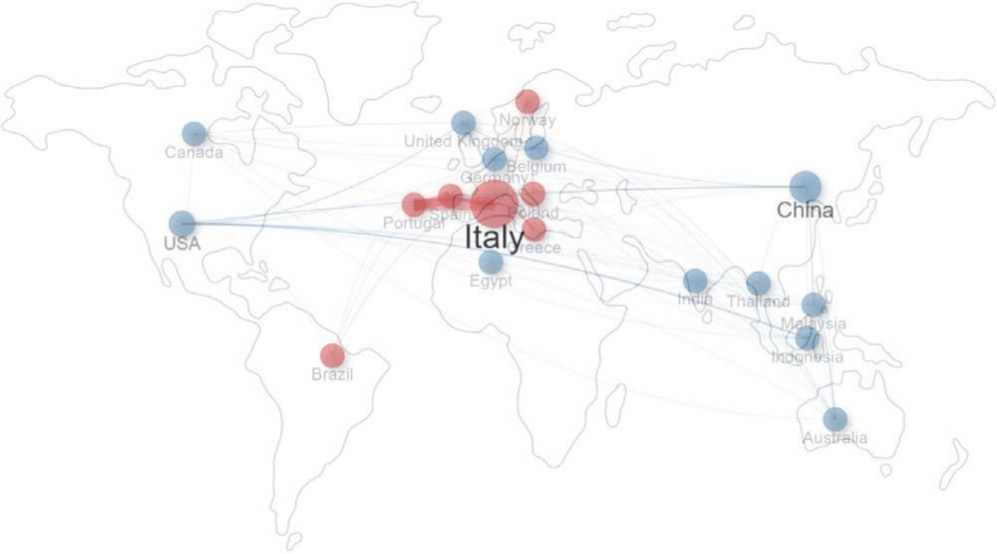
International collaboration network among countries based on co‐authorship in scientific publications. The size of the circle indicates the total number of publications, and the thickness of the lines indicates the frequency of collaboration.

Figure 7 illustrates the international collaboration network among countries based on co‐authorship in scientific publications. The figure attempts to reflect the geographic distance between countries; however, it is important to note that the map’s layout is only referential. Each node represents a country, with the size of the circle indicating the total number of publications attributed to that nation. Lines connecting the nodes represent collaborative links, with thicker lines denoting stronger or more frequent collaborations. The visualization reveals distinct clusters of collaboration, generally grouped by geographic or regional proximity.

The most prominent node in the network is Italy, which not only has one of the largest publication outputs but also serves as a central hub in the European cluster, forming strong collaborative ties with countries such as Portugal, Spain, Germany, and Poland. A separate cluster is dominated by China, also a major output contributor, with active collaboration particularly with Thailand, Malaysia, and Australia. The United States ranks third in terms of publication output, below the “Asian” group, and has strong collaboration with countries such as Indonesia and China.

Table 2 presents a bibliometric analysis of the 10 most cited scientific articles on the use of *Hermetia illucens* (black soldier fly larvae) in aquaculture across four distinct periods: 2007–2012, 2013–2018, 2019–2024, and 2025. During the first period (2007–2012), research was primarily exploratory, focusing on the nutritional potential of insect‐based feed ingredients and their impact on fish growth and chitin degradation. The most cited work in this phase, authored by Kroeckel et al. [25], examined growth performance in juvenile turbot, while other highly cited studies by St‐Hilaire et al. [27] addressed the use of fly prepupae as feed and the modulation of omega‐3 content via selected feed substrates. Normalized citation metrics in this period were consistent, indicating a stable rate of academic interest.

**Table 2:** Ten most cited research articles on the use of *Hermetia illucens* in aquaculture feed across the four time periods (2007–2025).

Number	Title	Author	Source	DOI	Total citations	TC per year	Norm TC	Year	Period
1	When a turbot catches a fly: evaluation of a prepupae meal of the black soldier fly (*Hermetia illucens*) as fish meal substitute, growth performance and chitin degradation in juvenile turbot (*Psetta maxima*)	Kroeckel et al. [25]	Aquaculture	10.1016/j.aquaculture.2012.08.041	407	29.1	1.00	2012	2007–2012
2	Fly prepupae as a feedstuff for rainbow trout, *Oncorhynchus mykiss*	ST‐Hilaire et al. [26]	Journal of the World Aquaculture Society	10.1111/j.1749‐7345.2006.00073.x	335	17.6	1.02	2007	2007–2012
3	Fish offal recycling by the black soldier fly produces a foodstuff high in omega‐3 fatty acids	ST‐Hilaire et al. [27]	Journal of the World Aquaculture Society	10.1111/j.1749‐7345.2007.00101.x	323	17	0.98	2007	2007–2012
4	Sensory analysis of rainbow trout, *Oncorhynchus mykiss*, fed enriched black soldier fly prepupae, *Hermetia illucens*	Sealey et al. [28]	Journal of the World Aquaculture Society	10.1111/j.1749‐7345.2010.00441.x	254	16.9	1.00	2011	2007–2012
5	Bioconversion of palm kernel meal for aquaculture: experiences from the forest region (Republic of Guinea)	Hem et al. [29]	African Journal of Biotechnology	10.4314/AJB.V7I8.58644z	38	2.1	1.00	2008	2007–2012
1	The potential of various insect species for use as food for fish	Barroso et al. [30]	Aquaculture	10.1016/j.aquaculture.2013.12.024	398	33.2	1.00	2014	2013–2018
2	Influence of different growing substrates and processing on the nutrient composition of black soldier fly larvae destined for animal feed	Tschirner and Simon [31]	Journal of Insects as Food and Feed	10.3920/jiff2014.0008	295	26.8	1.81	2015	2013–2018
3	Black soldier fly (*Hermetia illucens*) pre‐pupae meal as a fish meal replacement in diets for European seabass (*Dicentrarchus labrax*)	Magalhães et al. [8]	Aquaculture	10.1016/j.aquacultur.2017.04.021	294	32.67	2.61	2017	2013–2018
4	Defatted black soldier fly (*Hermetia illucens*) larvae meal in diets for juvenile Jian carp (*Cyprinus carpio var. Jian*): growth performance, antioxidant enzyme activities, digestive enzyme activities, intestine, and hepatopancreas histological structure	Li et al. [32]	Aquaculture	10.1016/j.aquaculture.2017.04.015	220	24.4	1.95	2017	2013–2018
5	Potential of insect‐based diets for Atlantic salmon (*Salmo salar*)	Belghit et al. [33]	Aquaculture	10.1016/j.aquaculture.2018.03.016	198	24.8	2.75	2018	2013–2018
6	The oil fraction and partially defatted meal of black soldier fly larvae (*Hermetia illucens*) affect differently growth performance, feed efficiency, nutrient deposition, blood glucose, and lipid digestibility of rainbow trout (*Oncorhynchus mykiss*)	Dumas et al. [34]	Aquaculture	10.1016/j.aquaculture.2018.03.038	170	21.3	2.36	2018	2013–2018
7	Influence of black soldier fly (*Hermetia illucens*) larvae oil on growth performance, body composition, tissue fatty acid composition, and lipid deposition in juvenile Jian carp (*Cyprinus carpio* var. Jian)	Li et al. [35]	Aquaculture	10.1016/j.aquaculture.2016.08.020	163	16.3	3.08	2016	2013–2018
8	Evaluation of black soldier fly (*Hermetia illucens*) larvae meal as partial or total replacement of marine fish meal in practical diets for Pacific white shrimp (*Litopenaeus vannamei*)	Cummins Jr et al. [36]	Aquaculture	10.1016/j.aquaculture.2017.02.022	161	17.9	1.43	2017	2013–2018
9	Effects of black soldier fly (*Hermetia illucens*) larvae meal protein as a fish meal replacement on the growth and immune index of yellow catfish (*Pelteobagrus fulvidraco*)	Xiao et al. [37]	Aquaculture Research	10.1111/are.13611	158	19.8	2.19	2018	2013–2018
10	Exploring the chemical safety of fly larvae as a source of protein for animal feed	Charlton et al. [38]	Journal of Insects as Food and Feed	10.3920/JIFF2014.0020	157	14.3	0.96	2015	2013–2018
1	Black soldier fly larvae meal can replace fish meal in diets of sea‐water phase Atlantic salmon (*Salmo salar*)	Belghit et al. [33]	Aquaculture	10.1016/j.aquaculture.2018.12.032	310	44.29	3.29	2019	2019–2024
2	Insect meals in fish nutrition	Nogales‐Mérida et al. [39]	Reviews in Aquaculture	10.1111/raq.12281	253	36.14	2.69	2019	2019–2024
3	Rainbow trout (*Oncorhynchus mykiss*) gut microbiota is modulated by insect meal from *Hermetia illucens* prepupae in the diet	Terova et al. [40]	Reviews in Fish Biology and Fisheries	10.1007/s11160‐019‐09558‐y	152	21.71	1.61	2019	2019–2024
4	High‐throughput sequencing of gut microbiota in rainbow trout (*Oncorhynchus mykiss*) fed larval and pre‐pupae stages of black soldier fly (*Hermetia illucens*)	Huyben et al. [41]	Aquaculture	10.1016/j.aquaculture.2018.10.034	140	20.00	1.49	2020	2019–2024
5	Substituting fish meal with *Hermetia illucens* in the diets of African catfish (*Clarias gariepinus*): effects on growth, nutrient utilization, haemato‐physiological response, and oxidative stress biomarker	Fawole et al. [42]	Aquaculture	10.1016/j.aquaculture.2019.734849	124	20.67	2.47	2019	2019–2024
6	The effects of dietary insect meal from *Hermetia illucens* prepupae on autochthonous gut microbiota of rainbow trout (*Oncorhynchus mykiss*)	Rimoldi et al. [43]	Animals	10.3390/ani9040143	120	17.14285714	1.27482679	2019	2019–2024
7	Evaluation of defatted black soldier fly (*Hermetia illucens* L.) larvae meal as an alternative protein ingredient for juvenile Japanese seabass (*Lateolabrax japonicus*) diets	Wang et al. [44]	Aquaculture	10.1016/j.aquaculture.2019.04.023	117	16.71428571	1.24295612	2019	2019–2024
8	Effects of black soldier fly (*Hermetia illucens* L.) larvae meal on growth performance, organs‐somatic indices, body composition, and hematobiochemical variables of European sea bass, *Dicentrarchus labrax*	Abdel‐Tawwab et al. [45]	Aquaculture	10.1016/j.aquaculture.2020.735136	115	19.16666667	2.286282306	2020	2019–2024
9	First insights on black soldier fly (*Hermetia illucens* L.) larvae meal dietary administration in Siberian sturgeon (*Acipenser baerii* Brandt) juveniles	Caimi et al. [46]	Aquaculture	10.1016/j.aquaculture.2019.734539	102	17	2.027833002	2020	2019–2024
10	A meta‐analysis of the effects of replacing fish meals with insect meals on growth performance of fish	Hua [47]	Aquaculture	10.1016/j.aquaculture.2020.735732	101	20.2	3.195820896	2021	2019–2024
1	Impact of defatted black soldier fly (*Hermetia illucens*) larvae meal on health, muscle texture, and intestinal microbiota in Pacific white shrimp (*Penaeus vannamei*)	Chang et al. [48]	Aquaculture	10.1016/j.aquaculture.2024.741755	2	2.00	7	2025	2025
2	Exploring the suitability of *Hermetia illucens* meal in *Clarias magur* catfish: effect on growth, physiology and flesh quality	Thomas et al. [49]	Journal of Insects as Food and Feed	10.1163/23524588‐00001089	1	1.00	3.5	2025	2025
3	Evaluation of black soldier fly oil as a replacement for fish oil in the diet of juvenile *Acanthopagrus schlegelii*: based on growth, apoptosis, and inflammation	Gu et al. [50]	Aquaculture	10.1016/j.aquaculture.2024.742005	1	1.00	3.5	2025	2025
4	Valorizing organic waste through black soldier fly larvae (*Hermetia illucens*): a sustainable solution for aquafeeds with key nutrients and natural bioactive polyphenols	Camperio et al. [51]	Sustainability	10.3390/su17051788	0	0.00	0	2025	2025
5	The defatted black soldier fly meal (*Hermetia illucens*) improved the pathogen resistance and gut health of Nile Tilapia (*Oreochromis niloticus*)	Wang et al. [52]	Fish and Shellfish Immunology	10.1016/j.fsi.2025.110242	0	0.00	0	2025	2025
6	Defatted black soldier fly (*Hermetia illucens*) diets improved hematoimmunological responses, biochemical parameters, and antioxidant activities in *Streptococcus iniae*‐infected Nile tilapia (*Oreochromis niloticus*)	Abd El‐Gawad et al. [53]	BMC Veterinary Research	10.1186/s12917‐025‐04484‐7	0	0.00	0	2025	2025
7	Carbohydrates in dietary ingredients for European seabass: impact on nutrient digestibility and waste production when reared in recirculating aquaculture systems	Syropoulou et al. [54]	Aquaculture	10.1016/j.aquaculture.2025.742182	0	0.00	0	2025	2025
8	Defatted black soldier fly meal in diets for juvenile pirarucu, *Arapaima gigas*: digestibility, growth performance, and health parameters	Gonçalves et al. [55]	Aquaculture	10.1016/j.aquaculture.2024.742071	0	0.00	0	2025	2025
9	Broad acceptance of sustainable insect‐based shrimp feeds requires reproducible and comparable research	Barth et al. [56]	Aquaculture International	10.1007/s10499‐024‐01769‐w	0	0.00	0	2025	2025
10	Black soldier fly meal as a gastrointestinal tract microbiota remodeling factor: a new natural and sustainable source of prebiotic substances for fish?	Rawski et al. [57]	Aquaculture Research	10.1155/are/8852384	0	0.00	0	2025	2025

Abbreviations: Norm TC, normalized total citation; TC, total citation.

In the subsequent periods (2013–2024), a notable increase in both the number of publications and their citation impact was observed, reflecting the growing relevance of insect meals in sustainable aquaculture. Research from 2013 to 2018 expanded to include detailed evaluations of nutrient digestibility, immune response, and feed efficiency across diverse fish species. This trend continued into the 2019–2024 period, with studies addressing advanced topics such as intestinal health, metabolic effects, and the complete replacement of fish meal. Several papers from the latest period demonstrated high normalized citation rates (e.g., >3.0), suggesting rapid uptake and influence within the academic community. In 2025, there appear to be fewer articles focused on topics such as fishmeal replacement but more on health parameters, muscle texture and quality, intestinal microbiota, immune response, and anti‐inflammation and antioxidation, indicating a shift towards the functional and health properties of BSFLM rather than its protein and lipidic value. Collectively, this progression underscores a shift from feasibility and fishmeal replacement studies to targeted investigations on the physiological, health, and ecological benefits of insect‐based aquafeeds.

**Figure 8:**
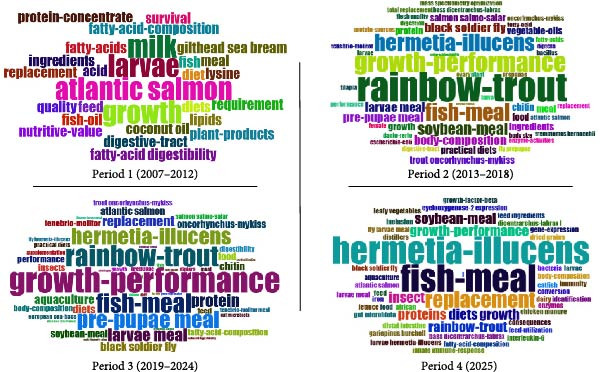
Word cloud visualization of keyword plus trends in research on black soldier fly (*Hermetia illucens*) in aquaculture across four time periods.

Figure 8 displays the evolution of research focus on the use of BSFLM in aquaculture, segmented into the four periods. Each quadrant presents a word cloud generated from Keywords Plus from each publication, with word size reflecting frequency or prominence in the literature.

Period 1 (2007–2012): Research during this early stage focused on traditional species such as Atlantic salmon and topics like larvae, fish meal, growth, and fatty acid digestibility. Alternative feed ingredients such as coconut oil and plant products began to appear, but *Hermetia illucen*s was not yet a prominent keyword.

Period 2 (2013–2018): This period marks the emergence of *Hermetia illucens* as a relevant topic, alongside growing interest in rainbow trout, growth performance, fishmeal replacement, and prepupae meal. Key themes included the role of soybean meal, chitin, and black soldier fly larvae in diets.

Period 3 (2019–2024): A significant increase in research activity is evident, with *Hermetia illucens*, growth performance, and rainbow trout dominating the discourse. Keywords such as insects, aquaculture, prepupae meal, and fatty acid composition reflect expanding investigations into insect‐based feed. Research interest in body composition, digestibility, and species‐specific effects also intensified.

Period 4 (2025–): This ongoing period shows a clear consolidation of *Hermetia illucens*, fishmeal, and replacement as core themes. The prominence of terms like diets, proteins, insects, soybean meal, and growth performance indicates a sustained focus on optimizing insect‐based feed formulations and understanding their impact on nutrition, physiology, and sustainability. Terms like gut microbiota, immune response, and gene expression suggest a growing interest in the functional and health effects of black soldier fly inclusion.

**Figure 9:**
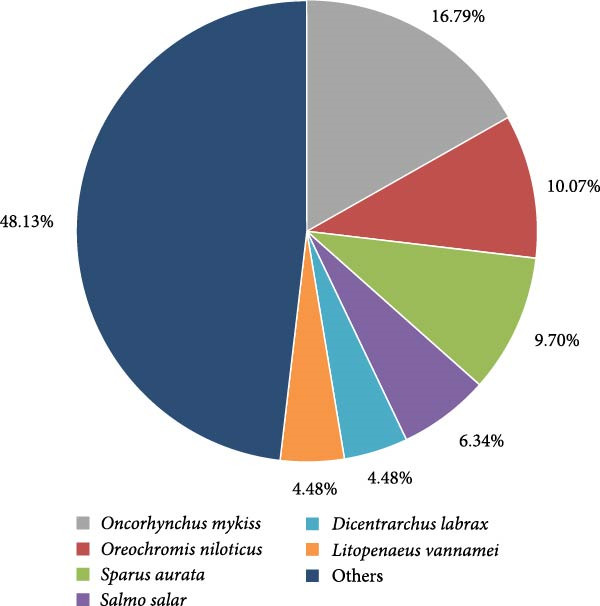
Frequency distribution of scientific names of species mentioned in research article titles in the total period (2007–2025).

Figure 9 presents the scientific names of species identified using an R script developed with the “stringr” library. The most frequently mentioned species was *Oncorhynchus mykiss*, which appeared in 16.79% of the cases, corresponding to 45 studies where it was the primary focus. Other notable species included *Oreochromis niloticus* and *Sparus aurata*, each respectively representing 10.07% and 9.70% of the total, followed by *Salmo salar* with 6.34%, and *Dicentrarchus labrax* and *Litopenaeus vannamei* with 4.48% each. The category labeled “Others” comprises 65 species, each accounting for less than 3% of the mentions but with a total of 48.13%. This group includes fish in the carp and catfish genus.

**Figure 10:**
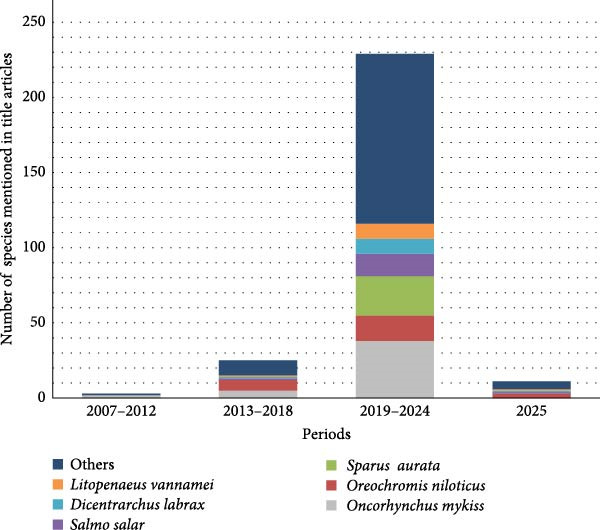
Relationship of species investigated during the four time periods.

Figure 10 illustrates the distribution of the main fish species mentioned in the titles of research articles across the four distinct time periods. In Period 1, only 2 species were identified: *Oncorhynchus mykiss*, appearing in two articles, and *Psetta maxima* in 1. During Period 2, the number of detected species increased to nine. The most frequently mentioned was *Oreochromis niloticus*, cited in seven studies, followed by *Oncorhynchus mykiss* with five mentions. Additionally, seven other species were identified in this period.

The highest species diversity was observed in Period 3, with a total of 66 species detected. In this period, *Oncorhynchus mykiss* once again emerged as the most studied species, appearing in 38 articles. It was followed by *Sparus aurata* (26 mentions), *Oreochromis niloticus* (17), and *Salmo salar* (15). At the same time, 62 other species were detected, many of them cited in a single article, indicating a broader but less frequent attention to other taxa.

Finally, in Period 4, a total of nine species were recorded. Despite the shorter time frame, *Oreochromis niloticus* remained prominent, appearing in three articles. Eight additional species were identified during this period with a single article each.

## 4. Discussion

### 4.1. Rationale for Using Keywords Plus

This scientometric analysis prioritized the use of Keywords Plus over Author Keywords. While Author Keywords are directly provided by the authors and reflect their intended focus, Keywords Plus are algorithmically generated based on recurrent terms in the titles of cited references, offering a broader contextual scope of the research topic. According to Chen et al. [58], Keywords Plus reveals latent thematic structures and improves the mapping of the intellectual base of a field, especially in emerging and interdisciplinary domains like BSFLM in aquaculture.

Additionally, a comparative study by Zhang et al. [24] evaluated the efficacy of Keywords Plus versus Author Keywords using scientific literature on patient adherence. The study found that although Keywords Plus is less comprehensive in reflecting an article’s specific content, it produces a broader range of terms that are more effective for mapping scientific fields and conceptual networks. Notably, Keywords Plus yielded more terms and broader descriptors than Author Keywords. This supports our rationale to employ Keywords Plus for its advantage in capturing overarching themes and interconnections that might be missed when relying solely on author‐selected terms.

### 4.2. Dominant Research Topics

The analysis of Keywords Plus across the four time periods revealed five recurring thematic clusters across the literature: “growth performance,” “rainbow trout,” “fishmeal,” “protein,” and “replacement.” These terms collectively reflect the core research directions in the application of BSFLM in aquaculture.

“Growth performance” is a key response variable in many nutrition trials and includes metrics such as weight gain, specific growth rate (SGR), feed conversion ratio (FCR), and protein efficiency ratio (PER). These indicators are essential for determining the biological efficacy and commercial viability of BSFLM as a dietary ingredient. Recent studies have demonstrated that BSFLM inclusion in aquafeeds can support a variety of growth and functional properties dependent on species and inclusion rate, with positive effects on growth, intestinal health, pathogen resistance, survival, antioxidative status, and immune response in various aquatic species, including rainbow trout, red seabream, tilapia, Atlantic salmon, European seabream, and white leg shrimp [1, 52, 32, 59–64].

“Rainbow trout” was identified as the most prevalent species keyword. This prominence can be attributed to two main factors: first, rainbow trout (*Oncorhynchus mykiss*) is among the most economically significant species in global aquaculture; and second, its physiology, nutritional requirements, and growth performance are extensively studied, making it a reliable model species for feed trials and comparative nutrition studies. This dual role, both commercial and academic, explains its centrality in BSFLM‐related research [65, 66].

The terms “fishmeal” and “protein” frequently co‐occur with “replacement” and “growth performance,” further reinforcing that the bulk of research focuses on evaluating BSFLM as a protein source capable of maintaining or enhancing performance while improving feed sustainability. Numerous studies have explored the nutritional profile of BSFLM, indicating it can serve as a viable alternative to conventional proteins due to its favorable amino acid composition and digestibility [39, 67].

“Replacement” emerged as a particularly frequent term, and although it is often associated with the substitution of fishmeal, it also reflects the broader effort to replace other traditional protein sources such as soybean meal. BSFLM meal offers not only a comparable protein profile but also the environmental advantage of being derived from organic waste, making it an attractive candidate for both fish meal and plant‐based protein replacement strategies [63, 66].

Overall, these dominant keywords illustrate the research community’s sustained focus on performance‐based evaluations and feed substitution strategies, with BSFLM positioned as a promising, versatile solution for both animal‐based and plant‐based protein challenges in aquaculture.

### 4.3. Countries and International Collaborations

The scientometric analysis revealed a broad and growing international interest in the application of BSFLM in aquaculture, marked by significant geographical diversity in both research productivity and collaboration. Italy emerged as the leading contributor in terms of TCs (TC = 2261), followed by the United States (1572), Norway (1480), and China (1293).

While countries such as Poland and Spain produced fewer total publications, they demonstrated strong performance in terms of average citations per article (57.9 and 55.3, respectively), indicating a high impact per study. Among the top performers, Norway (87.1), Nigeria (90.0), and Sweden (70.5) also stood out for their high citation averages, suggesting that even countries with moderate publication volumes are contributing influential research. These patterns reflect both national investment in scientific excellence and strategic focus areas within BSFLM–related research.

International collaboration was prominently visualized through co‐authorship networks, particularly among European countries, notably Italy, Belgium, Germany, and France, which demonstrated frequent regional and global partnerships. Italy showed strong bilateral ties with Portugal (*n* = 11), Spain (*n* = 6), and Germany (*n* = 3). Meanwhile, China exhibited extensive collaborations across Europe, Asia, and Africa, highlighting its role as a globally integrated research hub. Countries such as Malaysia, Indonesia, and India also engaged in diverse international collaborations, particularly with European and African partners.

These global patterns suggest that BSFLM research in aquaculture is not confined to a small number of nations but represents a distributed international effort involving high‐, middle‐, and low‐income countries. This is likely driven by the wide‐ranging relevance of BSFLM to food security, organic waste management, and sustainable animal feed development, issues of global significance.

A broader trend is also evident: developed countries generally produce more highly cited research than developing nations. This reflects differences in research infrastructure, funding availability, and access to high‐impact publication platforms. Such disparities are well documented across scientometric fields, where citation and output patterns often concentrate in wealthier countries, even when the subject matter, such as BSFLM, is globally applicable [68].

Furthermore, countries with large aquaculture sectors are often more active in BSFLM–related research, especially when sustainability and feed innovation are national priorities. China and Norway exemplify this alignment. However, the correlation is not absolute. For example, Indonesia and Vietnam, despite their large aquaculture industries, produce comparatively fewer BSFLM–focused publications, likely due to barriers such as limited research funding, publishing infrastructure, or international visibility [69].

Scientometric visibility in BSFLM research is shaped by a combination of industrial relevance, scientific infrastructure, and international collaboration. Countries that generate high‐impact research and maintain diverse collaborative networks are not only contributing to academic advancement but also shaping global innovation and knowledge exchange in the field of sustainable aquaculture.

The Sankey figure (Figure 3) provides an insightful visualization of authorial and geographical dynamics within the BSFLM research landscape. The diagram connects the top 20 most prolific authors (left) to their respective countries (middle) and the journals where their works are published (right). Notably, despite China ranking among the leading countries in overall publication output, none of its researchers appear among the top 20 global authors. This pattern suggests that BSFLM research activity in China may be broad but fragmented, distributed across multiple teams and institutions rather than concentrated within a few highly specialized research groups. In contrast, European countries such as Italy, Poland, Portugal, and Norway exhibit more cohesive and established networks of researchers, many of whom consistently publish in specialized aquaculture and animal nutrition journals such as Aquaculture, Aquaculture Nutrition, and the Journal of Insects as Food and Feed. This concentration of expertise likely contributes to the stronger visibility and influence of European authors in shaping global BSFLM research trends.

### 4.4. Temporal Evolution of Research Focus: 2007–2024

Segmenting the 10 most cited literature of the four distinct time periods (2007–2013, 2013–2019, 2019–2024, and 2025) reveals a clear evolution in research focus, species selection, thematic emphasis, and journal dissemination in the application of BSFLM in aquaculture.

During the earliest period (2007–2013), although the number of highly cited studies was relatively limited, the research was already strongly application‐driven. Contrary to initial assumptions of generality, studies during this phase prominently featured high‐value species such as rainbow trout (*Oncorhynchus mykiss*) and turbot (*Psetta maxima*). These early trials evaluated BSFLM inclusion in aquafeeds, focusing on growth performance, digestibility, chitin degradation, omega‐3 enrichment, and sensory quality of the fillets [25–29]. Keywords Plus for this period included “Atlantic salmon,” “growth,” “larvae,” “milk,” and “acid,” underscoring a combination of nutritional assessment and species‐specific investigations. Journal dissemination was spread across multiple platforms, with the Journal of the World Aquaculture Society, Aquaculture, and the African Journal of Biotechnology appearing among the most cited. This dispersion reflects the early exploratory nature of BSFLM research, before its centralization in mainstream aquaculture publishing.

The middle period (2013–2019) marked a notable transition toward nutritional validation and scaling of feed trials. Citation counts increased, and the literature became more concentrated around salmonid species, particularly Atlantic salmon (*Salmo salar*) and rainbow trout (*Oncorhynchus mykiss*), due to their commercial significance and common use in nutritional benchmarking, but additional species such as European seabass (*Dicentrarchus labrax*), Jian carp (*Cyprinus carpio* var. Jian), Pacific white shrimp (*Litopenaeus vannamei*), and yellow catfish (*Pelteobagrus fulvidraco*) were being evaluated [70, 71]. Research during this phase focused heavily on fishmeal replacement, protein quality, feed efficiency, and performance outcomes [8, 30–38]. Keywords Plus terms such as “rainbow trout,” “growth performance,” “fishmeal,” and “protein” demonstrate a clear emphasis on validating BSFLM meal as a functional substitute for conventional protein sources. Publication venues also began to consolidate, with Aquaculture Nutrition and Journal of Insects as Food and Feed emerging as key journals, reflecting increased disciplinary alignment and the rise of specialized platforms for insect‐based feed research.

In the following period (2019–2024), the volume of publications increased markedly, although average citations per paper were lower due to the shorter citation window. This phase exhibited the greatest thematic diversity based on the scope of study content, with research extending beyond salmonids to include tilapia (*Oreochromis niloticus*), catfish (*Clarias gariepinus*), carp (*Cyprinus carpio*), Siberian sturgeon (*Acipenser baerii*), and European sea bass (*Dicentrarchus labrax*), while rainbow trout remained a central model species. Studies during this phase increasingly investigated immune responses, microbiome modulation, and sustainability metrics, signaling a gradual shift from solely performance‐based validation to more integrative assessments [6, 7, 42, 45–47, 72–75]. However, the Keywords Plus remained anchored around terms such as “growth performance,” “rainbow trout,” “fishmeal,” and “digestibility,” reflecting a continued emphasis on protein replacement efficacy and species performance. These terms suggest that while conceptual diversification is occurring within article content, citation‐linked keyword structures still highlight the foundational research priorities. A significant trend during this phase was the centralization of high‐impact publications in Aquaculture, underscoring the consolidation of BSFLM research within the core of aquaculture science.

In the final period (2025), only 15 articles were published since accessing Scopus and WOS (March 25, 2025) with a TC of 4, both reasonable numbers considering the short timeframe. Species diversification remained high with articles investigating the use of BSFLM in diets for shrimp (*Litopenaeus vannamei*), catfish (*Claria smagur*), tilapia (*Oreochromis niloticus*), European seabass (*Dicentrarchus labrax*), pirarucu (*Arapaima gigas*), Atlantic salmon (*Salmo salar*), carp (*Cyprinus carpio*), and black seabream (*Acanthopagrus schlegelii*). While studies on growth performance, digestibility, and fish meal and oil replacement are still prevalent, there is a continuing observable shift towards more precise and functional performance metrics such as muscle texture, intestinal microbiota, physiology, immunological responses, apoptosis and inflammation, polyphenols, and waste valorization [48–55, 76–79]. Keywords Plus for this year remain very similar to previous time periods, with emphasis on “fishmeal,” “growth performance,” “replacement,” “digestibility,” “rainbow trout,” and “soybean meal.” The journal Aquaculture remains the primary high‐impact journal where articles for 2025 are published.

Overall, the progression across these four periods illustrates the maturation of BSFLM research from early proof‐of‐concept studies to species‐specific feed validation and ultimately to more diverse and sustainability‐conscious applications. This trajectory reflects not only scientific refinement but also growing industry relevance and policy interest, supported by broader species inclusion, methodological diversity, and consistent publication in leading aquaculture journals. The shift of BSFLM towards functional topics such as health, immunity, and antioxidation reflects not only a research trend but also a potential competitive advantage of this novel feed component.

### 4.5. Temporal Changes and Frequency of Species Being Investigated

Figure 9 illustrates the species distribution in aquaculture‐focused BSFLM research, revealing that while the literature spans a range of organisms, it remains heavily concentrated on a few key species. *Oncorhynchus mykiss* accounts for 16.79% of publications, followed by *Oreochromis niloticus* (10.07%) and *Sparus aurata* (9.70%). Lesser but still notable representation is seen for *Salmo salar*, *Dicentrarchus labrax*, and *Litopenaeus vannamei*, each contributing 6.34%, 4.48%, and 4.48%, respectively. Interestingly, almost half (48.13%) of all studies fall into the “Other” category, which points to a growing interest in diverse or regionally important species outside the commonly studied finfish and crustaceans. Carps, despite being the globally dominant aquaculture group with 31.8 million tonnes in 2022 (51.6% of finfish production), remain relatively underexplored in BSFLM research [69]. Our dataset includes six carp species with a total of 13 publications, positioning them between *Salmo salar* and *Dicentrarchus labrax* in publication frequency. This discrepancy underscores a research gap given the global importance of carps in food security, especially in Asia. This same disparity can be applied to catfish research within the database used for this manuscript.

Production data from FAO further highlight the imbalance between global aquaculture output and BSFLM research focus [69]. In 2022, salmonids and seabreams represented only 6.9% and 0.9% of global finfish production, respectively, while penaeid shrimp dominated crustacean farming with 62.2% of global output. Cichlids, including *Oreochromis* spp., accounted for 10.6% of global finfish production. Most notably, carps alone constituted 51.6%, the majority share of finfish aquaculture worldwide. Despite this, carps and cichlids, which together make up over 62% of global finfish production, receive disproportionately limited attention in BSFLM research, underscoring a significant disconnect between production realities and current research priorities. This misalignment is further reflected in geographic patterns. As shown in Figure 5, Europe dominates BSFLM aquaculture publications (50%), despite representing only 3.2% of global aquaculture production in 2022 [69]. In contrast, Asia, which contributes nearly 90% of global production, is the source of just 30% of the literature. Countries like China, India, Indonesia, and Vietnam, which collectively lead in carp, tilapia, and shrimp production, are relatively underrepresented in terms of BSFLM publication output. These trends suggest that BSFLM research is more closely tied to institutional capacity and research funding availability than to global production realities.

However, there is also a regional alignment between species dominance and research priorities. For instance, rainbow trout and Atlantic salmon are extensively farmed in Europe and North America, where research output on BSFLM in these species is concentrated. Similarly, BSFLM studies on tilapia and shrimp are often conducted in China, Indonesia, Thailand, and Vietnam, countries with major production and consumption of these species. This indicates that while global alignment may be lacking, localized research efforts do tend to reflect regional aquaculture practices and market relevance.

The temporal dynamics in Figure 10 further illustrate the trajectory of BSFLM research across species and time. Between 2019 and 2024, publication activity surged both in volume and taxonomic breadth. This period aligns with heightened interest in sustainable feed alternatives, yet most of the increased research continued to favor species predominant in high‐income or high‐publishing regions. Although recent years have introduced greater diversity in species investigated, the dominant taxa remain salmonids, seabreams, tilapia, and shrimp, species central to aquaculture industries in Europe, Southeast Asia, and Latin America.

## 5. Conclusion

This scientometric study offers a detailed overview of the evolving research landscape surrounding the use of black soldier fly larvae (*Hermetia illucens*) in aquaculture. From early experimental trials to contemporary investigations into functional and physiological outcomes, the field has expanded considerably in volume, species coverage, and thematic scope. A notable transition is evident from initial studies focused on protein replacement toward a broader exploration of BSFLM’s role as a functional feed ingredient with health‐promoting properties. However, the analysis also reveals a persistent disconnect between global aquaculture production and publication output. Research remains disproportionately centered on salmonids, red seabream, and European seabass, species with limited global production, while carps and catfish, which dominate global finfish output, are comparatively underrepresented. This disparity may be attributed to the fact that cost‐effective carp and catfish feeds are typically formulated without expensive BSFLM to maintain low production costs, whereas diets for salmonids, seabream, and seabass can more readily absorb such price increases, an observation that warrants further economic investigation. Additionally, Europe accounts for most BSFLM–related publications despite producing a small share of the world’s farmed fish, whereas Asia, which leads in aquaculture production, contributes relatively fewer studies. This imbalance appears driven by a concentration of research efforts in high‐income countries and a focus on regionally favored species. To enhance the global relevance and impact of BSFLM research, future efforts should prioritize underrepresented species and bolster cross‐regional collaboration. Aligning research priorities with aquaculture realities will be crucial to realizing the full potential of insect‐based feeds in advancing sustainable and resilient food systems. Based on these findings, future research should focus on improving coherence and global alignment in BSFLM research by: (1) consolidating studies across the wide range of carp and catfish species to generate more comparable and regionally relevant data; (2) fostering coordinated international networks that link the high research capacity of Europe and North America with the large‐scale production systems of Asia; (3) integrating economic and life‐cycle assessments to evaluate the scalability and cost‐effectiveness of BSFLM under commercial conditions; (4) advancing studies on the functional bioactive compounds of BSFLM, such as polyphenols, antimicrobial peptides, and chitin for their potential roles in immunity, antioxidant capacity, and gut microbiota modulation. These efforts will help reduce research fragmentation, enhance practical application, and support the development of globally relevant, sustainable aquafeed strategies.

## Conflicts of Interest

The authors declare no conflicts of interest.

## Funding

The authors did not receive support from any organization for the submitted work.

## Supporting Information

Additional supporting information can be found online in the Supporting Information section.

## Supporting information


**Supporting Information** Supporting information is the raw database of the analyzed scientific articles.

## Data Availability

The data that support the findings of this study are available from the corresponding author upon reasonable request.
